# Deep in the Jelly: Histochemical and Functional Aspects of Mucilage-Secreting Floral Colleters in the Orchids *Elleanthus brasiliensis* and *E. crinipes*

**DOI:** 10.3389/fpls.2019.00518

**Published:** 2019-04-24

**Authors:** Fábio Cassola, Carlos Eduardo Pereira Nunes, Makeli Garibotti Lusa, Vera Lúcia Garcia, Juliana Lischka Sampaio Mayer

**Affiliations:** ^1^Institute of Biology, State University of Campinas, Campinas, Brazil; ^2^Department of Organic and Pharmaceutical Chemistry, Chemical, Biological and Agricultural Pluridisciplinary Research Center, Paulínia, Brazil; ^3^Department of Biological and Environmental Sciences, University of Stirling, Stirling, Scotland; ^4^Center of Biological Sciences, Federal University of Santa Catarina, Florianópolis, Brazil

**Keywords:** Atlantic Forest, Epidendroideae, histochemistry analysis, microstructure, plant anatomy and morphology, secretory structure, Orchidaceae

## Abstract

Colleters are trichomes or emergencies that produce a sticky exudate consisting of a mixture of mucilage, lipids, terpenes, and phenolic compounds. Colleters occur in at least 60 families of angiosperms; however, reports of them are scarce for the Orchidaceae. *Elleanthus brasiliensis* is distinguished by the presence of an abundant gelatinous secretion that covers almost all of its inflorescences. We aimed to describe the histology of colleters in inflorescences of *E. brasiliensis* and *Elleanthus crinipes*, and to analyze the chemical composition of their secretion to better understand the functions of these secretory structures. Due to the low frequency of colleters and lack of visible secretion in *E. crinipes*, histochemical tests and chemical analyses were not performed for this species. Colleters are of a brush type and their secretion has, at the same time, hydrophilic and lipophilic components. Histochemical tests further revealed the presence of pectin, mucilage, lipids, terpenes, phenolic compounds, and proteins. The GC-MS analysis confirmed the presence of γ-sitosterol and palmitic, linoleic, and stearic acids in the secretion of *E. brasiliensis*. Infrared analysis indicated the possible presence of polysaccharides in the secretion. The occurrence of colleters in both species studied and in other orchids described in the literature suggests that these structures are common in the inflorescences of tropical orchids. In these environments, the hydrated polysaccharides in the secretion form a dense matrix that can act as a physical barrier, and terpenes may help to protect against herbivores and pathogenic microorganisms. This information broadens our knowledge of the morphological and chemical diversity of the secretions produced by orchid colleters.

## Introduction

Plant secretions are synthesized and eliminated by specific cells, which can occur in isolation or form differentiated glandular structures, such as trichomes, emergencies, canals, cavities, and laticifers (Castro and Demarco, 2008; Castro and Machado, 2013). Among such differentiated glandular structures, colleters are emergencies (Leitão and Cortelazzo, 2008) formed of epidermal and subepidermal tissues or of trichomes (Mayer et al., 2011; Machado and Rodrigues, 2013) originating only from the protoderm, which produce a sticky exudate composed of mucilage and/or lipid-like substances (Fahn, 1979; Mayer et al., 2013; Machado et al., 2015; Capelli et al., 2017). In general, this secretion is associated with vegetative and reproductive organs in the process of differentiation, and can protect them against dehydration or attacks by herbivores and microorganisms (Whittier and Peterson, 1984; Thomas, 1991; Mayer et al., 2013; Coutinho et al., 2015; Lusa et al., 2015).

In eudicots plants, the most common types of colleters are composed of secretory palisade epidermal cells, with the central axis formed by parenchyma and in some cases including vascular bundles (Miguel et al., 2006; Castro and Machado, 2013). In monocots plants, these structures can be trichomes, bulky cells with a dense cytoplasm and atrophied nucleus, hairs, or epidermal appendages (Leitão and Cortelazzo, 2008; Mayer et al., 2011). Despite this distinction, many aspects of the secretion, physiology, anatomy, and ultrastructure of colleters are poorly known due to a lack of comprehensive and in-depth studies on them (Fahn, 1979; Macêdo et al., 2016).

**FIGURE 1:**
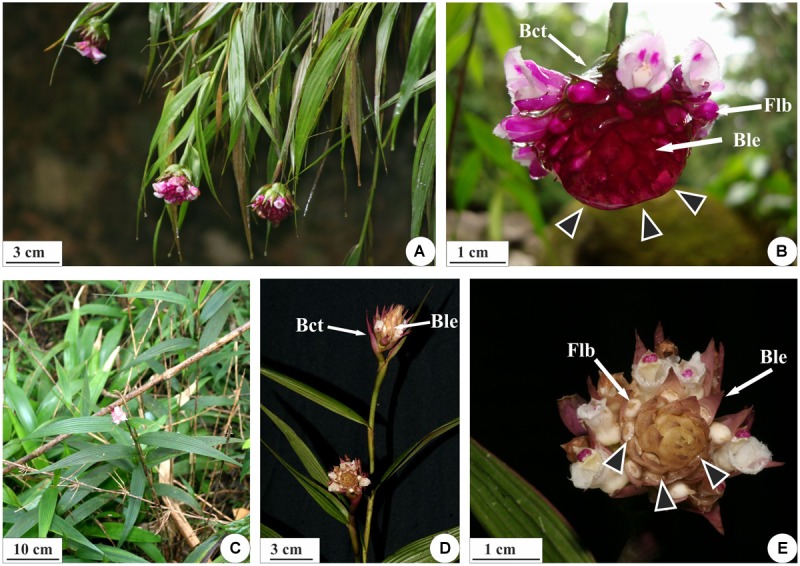
Habit and inflorescences of *E. brasiliensis* **(A,B)** and *E. crinipes* **(C–E)**. **(A)** Pending branches of *E. brasiliensis* with terminal inflorescences. **(B)** Detail of the inflorescence enveloped at the base by bracts, with basal flowers in anthesis and a large amount of secretion (arrowheads) covering the floral buds. **(C,D)** Erect branches of *E. crinipes* with terminal inflorescences. **(E)** Detail of the inflorescence with basal flowers in anthesis and a small quantity of secretion overlapping the flower buds (arrowheads). Bct, bracts; Ble, bracteole; Flb, flower buds.

The genus *Elleanthus* C. Presl contains 150 described species, with representatives in Central and South America, and its greatest diversity occurs in the Andes (Dudek et al., 2017). *Elleanthus brasiliensis* (Lindl.) Rchb. f. is found in the humid forests of eastern Brazil and the Guianas, while *Elleanthus crinipes* Rchb. f. is endemic to southeastern Brazil, where it is found in highland forests in different physiognomies of the Atlantic Forest (Nunes et al., 2016). *E. brasiliensis* stands out from other species due to the large amount of mucilaginous secretion that covers its inflorescences, refracting the reddish color of the bracts and giving the globular inflorescence a bright appearance (Figure 1A,B). In *E. crinipes* (Figure 1C), this secretion is conspicuously scarce (Figure 1D,E), although these two species may occur in the same environments and share the same pollinators (Nunes et al., 2013, 2016).

Knowledge of the secretory structures of plants and the chemical constitution of their secretions can help elucidate the relationships between patterns and processes in the ecology of interactions involving plants. Thus, this study aimed to identify and describe the secretory structures present in the inflorescences of *E. brasiliensis* and *E. crinipes* and analyze the chemical composition of their secretion to estimate its functional role in the analyzed species and provide useful data for ecological, taxonomic, and chemosystematic purposes.

## Materials and Methods

### Plant Material

*Elleanthus brasiliensis* and *E. crinipes* are epiphytic or rupicolous herbs that occur in environments with high air humidity (Nunes et al., 2016). *E. brasiliensis* has pendent stems (Figure 1A), while *E. crinipes* has erect stems (Figure 1C), at the apex of which reproductive buds give rise to racemose inflorescences (Figure 1B,D,E). Due to the low frequency of colleters and the lack of visible or abundant secretion in *E. crinipes*, histochemical tests were not performed for this species. In despite of belonging to the same genus, these species are not closely related inside *Elleanthus* and are classified in distinct sections within it (Dressler, 2006).

Samples were collected during the flowering and fruiting seasons from 2012 to 2015. Sampling was carried out at two sites, a lowland site (less than 100 m a.s.l.) and a highland one (800–1000 m a.s.l.), both in areas of the Atlantic Forest (Ombrophilous Dense Forest; Veloso et al., 1991) in Serra do Mar State Park (SMSP), southeastern Brazil. Inflorescences of *E. brasiliensis* were collected in the lowland area where this species is more abundant, in the municipality of Ubatuba, São Paulo state (23°20021.9′ S, 44°50014.5′ W). Inflorescences of *E. crinipes* were collected in the highland area, between the municipalities of São Luiz do Paraitinga, Cunha, and Natividade da Serra, São Paulo state (23°26008′ S, 45°13022.5′ W and 23°19055′ S, 45°05049′ W, respectively). Vouchers (12/02/2010, *E. brasiliensis*, C.E.P. Nunes 01; *E. crinipes*, C.E.P. Nunes 02) are deposited in the herbarium of the University of Campinas “Prof. Dr. João Semir” (UEC).

### Light and Scanning Electron Microscopy

To analyze the general structure of colleters, tissue samples of flowers and floral bracts at different developmental stages were fixed in a formalin–acetic acid–alcohol (FAA) solution for 24 h (Johansen, 1940) and in Karnovsky’s solution for 48 h (Karnovsky, 1965), and were then subjected to reduced pressure to allow adequate penetration by the fixative. Samples were subsequently stored in 70% (v/v) ethanol. The material was then dehydrated through a tertiary butanol series (Johansen, 1940). One part of the samples was embedded in plastic resin (Leica Historesin^®^, Heraeus Kulzer, Hanau, Germany), while another part was embedded in Paraplast^®^X-tra (Fisher, cat. n° 23-021-401) (Johansen, 1940). The embedded samples were longitudinally and transversely sectioned with a rotary microtome (Leica^®^) equipped with a type C blade. For the samples in Paraplast^®^, sections were cut at a thickness of 12 μm, had the paraffin removed, and were then stained with safranin O and astra blue (Srebotnik and Messner, 1994). For the samples in Historesin^®^, sections were cut at a thickness of 5–7 μm and stained with 0.05% toluidine blue at in a citrate-phosphate buffer with a pH of 4.5 (Sakai, 1973). After staining, the glass slides were mounted with the synthetic resin Entellan^®^(Merck^®^). The serial sections were examined microscopically (Olympus BX51) under polarized light to verify the occurrence of starch grains, crystals, and lignified cell walls. Images of inflorescences were taken in the field with a digital camera (Canon EOS20D).

For scanning electron microscopy (SEM), flower samples were fixed as described by Karnovsky (1965) for 24 h (modified by preparation in pH 7.2 phosphate buffer), dehydrated in a graded ethanol series, and subjected to critical point drying with CO_2_ (Horridge and Tamm, 1969). Samples were then attached to aluminum stubs and coated with gold (30–40 nm). Finally, the samples were examined under a LEO model VP 435 scanning electron microscope (SEM) at 10 kV.

### Histochemical Analysis

Several different histochemical procedures were carried out to detect the main classes of chemical compounds typically produced by plant secretory structures. The histochemical reactions used comprised the following: reaction with coriphosphine under fluorescence to test for pectins (Ueda and Yoshioka, 1976); ruthenium red for mucilage and pectic substances (Johansen, 1940); Sudan III, Sudan IV (Jensen, 1962), and Sudan black B (Pearse, 1968) for total lipids; Nile blue sulfate for neutral lipids (Cain, 1947); aniline blue black for proteins (Fisher, 1968); Nadi reagent for terpenes (David and Carde, 1964); and ferric chloride for phenolic compounds (Johansen, 1940). Sections were examined immediately after each reaction under an Olympus^®^BX 51 microscope. Photomicrographs were taken of the samples under the Olympus^®^BX 51 microscope, which was equipped with an Olympus DP 71 camera. For the analysis of the reaction with coriphosphine, the same microscope was used, and was equipped for epifluorescence illumination with a U-LH100HG mercury lamp to provide excitation (bandpass filter: 450–490 nm) and suppression (long-pass filter: 515 nm). Control sections were prepared simultaneously to the histochemical tests, in accordance with standard procedures. To verify the natural appearances of organs and secretions, untreated sections were prepared and observed. Images were recorded from light and epifluorescence microscopy by capturing images of the slides using an Olympus DP71 video camera, coupled to the abovementioned microscope.

### Ethanolic Extract Preparation

The secretion from the colleters was collected directly from the surfaces of inflorescences of *E. brasiliensis* (the species with abundant secretion) containing floral buds using a Pasteur pipette, and was then frozen. Four to five inflorescences (13.2 mg of secretion) were used from three different individuals. Due to the high-volume reduction of the material when dry, it was necessary to join several individuals for these analyses. To obtain the ethanolic extract of the secretion, each sample was thawed, 15 mL of ethanol was added to it, and it was then placed in an ultrasonic bath for 20 min. The solution was dried under reduced pressure, which yielded 2.96 mg of the ethanolic extract.

### Gas Chromatography Coupled to Mass Spectrometry (GC-MS)

The GC-MS analysis were performed on an Agilent^®^6890N chromatograph, with a 5975-mass detector and a 7683B automatic injector coupled to a HP5MS capillary column (30 m × 0.25 mm × 0.25 μm). The conditions of this analysis were as follows: the temperature of the injector was set at 220°C, the detector was set at 280°C, and the column was set at 60°C and increased by 3°C/min up to 240°C, and then maintained at 240°C for 7 min.; and He super dry was added at 1.0 mL/min as the carrier gas. The mass spectra obtained for each signal observed in the chromatogram were compared to the fragmentation patterns of the NIST 2005 library compounds of the equipment with a searching similarity of ≥90%.

### Methylation of the Extract

Two milliliters of dichloromethane and 0.5 mL of diazomethane solution in ethyl ether were added to 1.4 mg of the ethanolic extract of the mucilaginous secretion. After evaporation of all of the solvent, the sample was resuspended with 1 mL of ethyl acetate and injected into the GC-MS system.

### Infrared Spectroscopic Analysis

Infrared reflectance spectra were obtained in an infrared spectrometer with Fourier Transform (IR-TF) in the region of 4000–450 cm^−1^. An attenuated total reflectance accessory was used in the Cary 630 – FTIR Spectrometer (Agilent Equipment). To make the sample suitable for this analysis, a potassium bromide (KBr) tablet was prepared from 7.9 mg of a dry sample of the jelly secretion.

**Table 1:** Classes of substances evidenced, and their reagents used in *E. brasiliensis* colleters and its secretion.

Substance class	Reagents	Result of reaction^∗^
Pectins	Coriphosphine	+++
Mucilage and pectic substances	Ruthenium red	+++
Total lipids	Sudan III	++
Total lipids	Sudan black B	+++
Neutral lipids	Nile blue sulfate	+++
Terpenes	Nadi reagent	++
Phenolic compounds	Ferric chloride	++
Proteins	Aniline blue black	+

*^∗^The intensity of reactions was indicated by: +++ (very intense reaction); ++ (intense reaction); + (little intense reaction).*

## Results

### Structural and Histochemical Characterization of the Colleters

**FIGURE 2:**
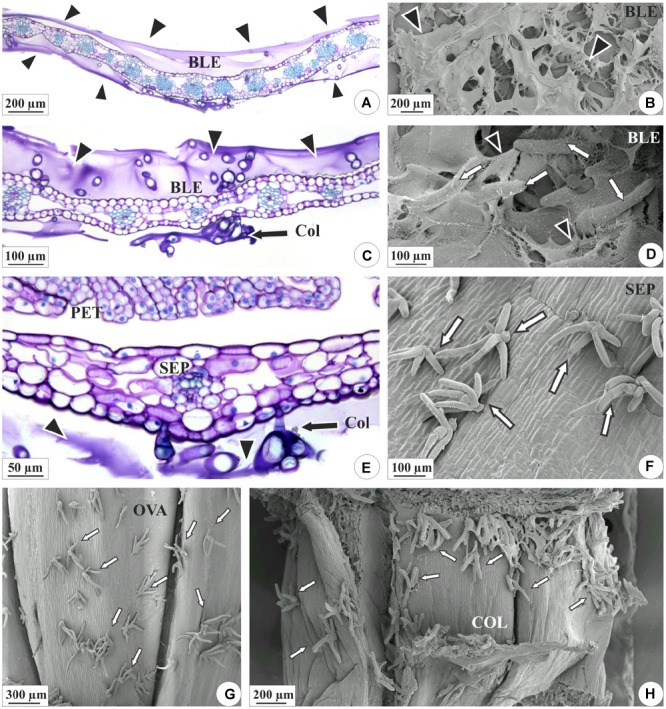
Distribution of the colleters (arrows) and secretion in the floral organs of *E. brasiliensis*. **(A,C,E)** Light microscopy. **(B,D,F–H)** Scanning electron microscopy. **(A–D)** Bracteole covered by a large amount of secretion (arrowheads). **(C,D)** Magnified detail of the colleters (“Col” and white arrows) involved in secretion. **(E,F)** Sepals presenting colleters and a lower amount of secretion. **(G)** Ovarian wall showing colleters. **(H)** Floral column with colleters on the surface. BLE, bracteole; COL, floral column; OVA, ovary; PET, petal; SEP, sepal.

**FIGURE 3:**
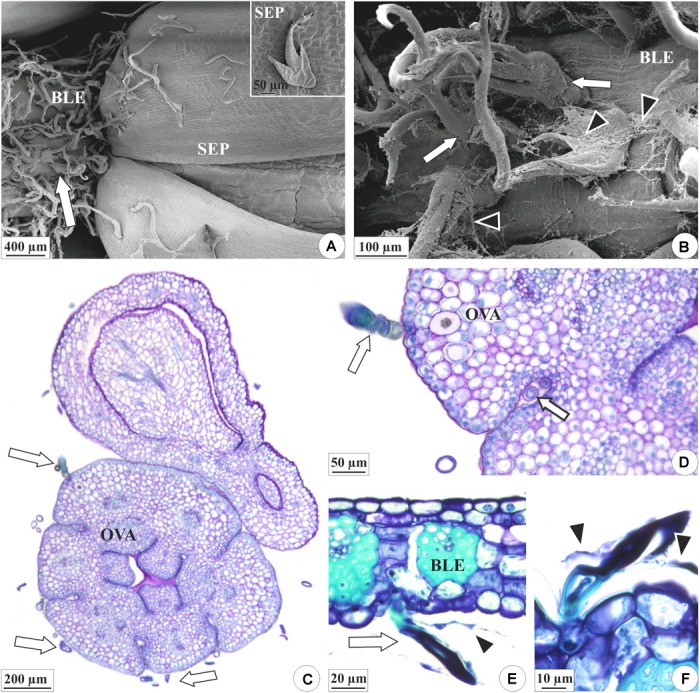
Distribution of the colleters (arrows) and secretion in the floral organs of *E. crinipes*. **(A,B)** Scanning electron microscopy. **(C–F)** Light microscopy. **(A)** Floral bud with bracteole showing colleters (white arrow) and sepals surrounding the bud. Inset: colleter on the surface of the sepal. **(B)** Magnified detail of the bracteole, showing colleters, and secretion (arrowheads). **(C)** Ovary with colleters on outer surface, as viewed from above the ovary axis of a lateral flower surrounded by a bract. **(D)** Detail of the ovary, showing colleter on the surface and the recess of the wall (white arrow). **(E)** Bracteole with a colleter. **(F)** Detail of the bracteole’s colleters surrounded by their secretion. BLE, bracteole; OVA, ovary; SEP, sepal.

The axis of each inflorescence was found to be surrounded by fibrous bracts, and each floral bud is in turn encased by equally rigid bracts (Figure 1B,D,E). In the reproductive buds and mature inflorescences of both species, the colleters produce a sticky secretion, which envelopes the floral buds and external parts of the flowers (Figure 1B,E). These secretory structures are present in the both surfaces of the bracts, bracteoles, sepals and on ovary wall, and floral column in *E. brasiliensis* (Figure 2A–H) and are very abundant, especially in the bracteoles (Figure 2A–D), which would explain the more abundant secretion by this species (Figure 2A–D). In *E. crinipes*, colleters are also present in the bracts, bracteoles (Figure 3A,B,E,F), sepals (Figure 3A), and ovary wall (Figure 3C,D). However, their frequency is remarkably lower when the median region is analyzed. On five bracts of each species in an area of 141 mm^2^ were found on average of 2.4 colleters in *E. crinipes* and 18 colleters in *E. brasiliensis*. In *E. crinipes* the colleters occur only in the median region, whereas in *E. brasiliensis* these structures are present throughout the bract. This characterizes the difference in the amount of secretion produced by these species (Figure 1E, 3B,E,F).

**FIGURE 4:**
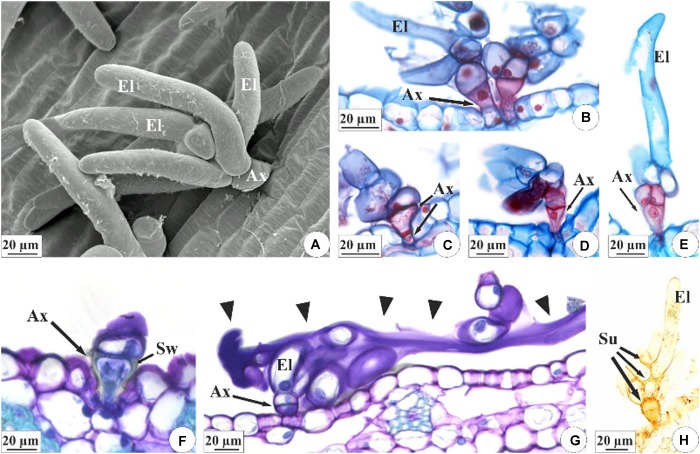
Characterization of the colleters of *E. brasiliensis* **(A–E,G,H)** and *E. crinipes* **(F)**. **(A)** Scanning electron microscopy. **(B–H)** Light microscopy. **(A)** Brush-type colleter with short basal axis and elongated terminal cells. **(B–E)** Colleter with short cup-shaped basal axis composed of one **(B)**, two **(C,D)**, or three cells **(E)**, and presenting lignified and suberified secondary wall deposition, as indicated by double-staining with afstra blue and safranin. Elongated cells have only primary cell walls. **(F)** Detail of the basal axis of the colleter showing the cup-shaped cell with a secondary wall. **(G)** Colleter with elongated cells releasing secretion (arrowheads) that covers the surface of the bracteole. **(H)** Histochemical test with Sudan IV indicating suberification in the cells of the colleter’s axis and in the cell walls of the elongated cells that connect with the cells of the axis. Ax, axis of colleter; El, elongated cell; Sw, secondary wall; Su, suberized wall.

The colleters of *E. brasiliensis* are of a brush type (Figure 4A–E,G,H), each containing a short, cup-shaped shaft at the base composed of one to three cells (Figure 4B–E). In the terminal part, these structures bear three to seven elongated cells (Figure 4A,B,E). The axillary cells present lignified and suberified secondary wall deposition (Figure 4B–F,H). However, the elongated cells present only primary cell walls (Figure 4B–E) and constitute the site of secretion synthesis (Figure 4G). On the other hand, the walls of the elongated cells in connection with the axis cells also exhibit suberification (Figure 4H). The structure of colleters of *E. crinipes* is similar to that described for the colleters of *E. brasiliensis*, as seen in the Figure 3B,E,F. Nevertheless, the basal shaft is more elongated in *E. crinipes* (Figure 3B).

**FIGURE 5:**
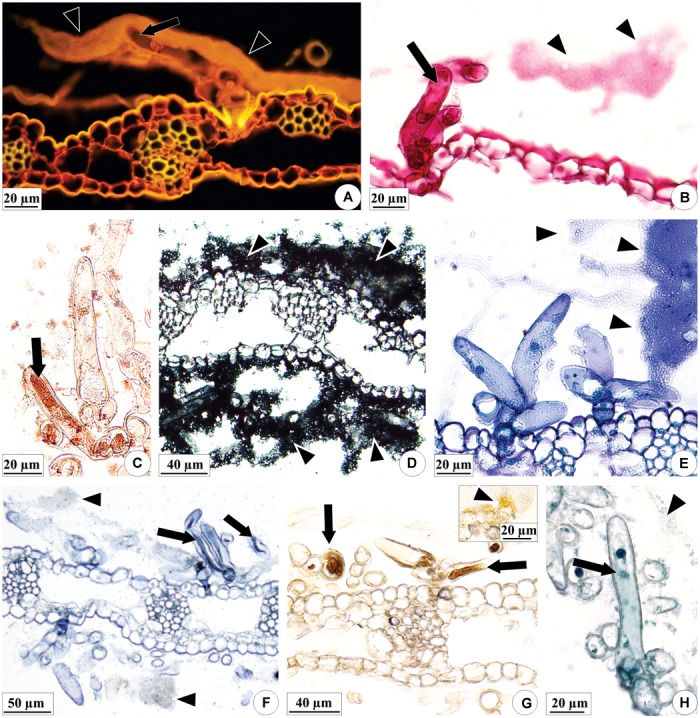
Histochemical characterization of the colleters (arrows) of *E. brasiliensis*. **(A)** Pectic substances present in elongated cells, and abundant in the secretion of the colleter. **(B)** Mucilage and pectic polysaccharides in the protoplast and secretion of an elongated cell. **(C)** Total lipids present in the elongated cell protoplast. **(D)** Total lipids present in the secretion on the bracteole. **(E)** Neutral lipids present in the secretion of the colleter. **(F)** Terpenoids within elongated cells, and also less evidently present in the secretion. **(G)** General phenolic compounds in elongated cells. **(H)** Proteins in the protoplasts of elongated cells and poorly evident in the secretion. Histochemical reactions: coryphosphine **(A)**, ruthenium red **(B)**, Sudan III **(C)**, Sudan black B **(D)**, Nile blue sulfate **(E)**, Nadi reagent **(F)**, ferric chloride **(G)**, and aniline blue black **(H)**. Arrowheads: secretion; black arrows: substances present in elongated cells.

The content of the colleters of *E. brasiliensis* and the secretion produced by these structures are hydrophilic and lipophilic in nature (Table 1 and Figure 5A–H). In general, lipophilic substances and phenolic compounds are evident in the secretory cell vacuoles (Figure 5C,F,G, black arrows), and the hydrophilic substances occupy the entire protoplast (Figure 5B). The exudate is a mixture of these hydrophilic and lipophilic substances (Figure 5, arrowheads), containing pectic substances (Figure 5A), mucilage (Figure 5B, black arrow), lipids (Figure 5D,E), terpenoids (Figure 5F), phenolic compounds (Figure 5G), and proteins (Figure 5H).

### Chemical Analysis of the Secretion From the Colleters

**FIGURE 6:**
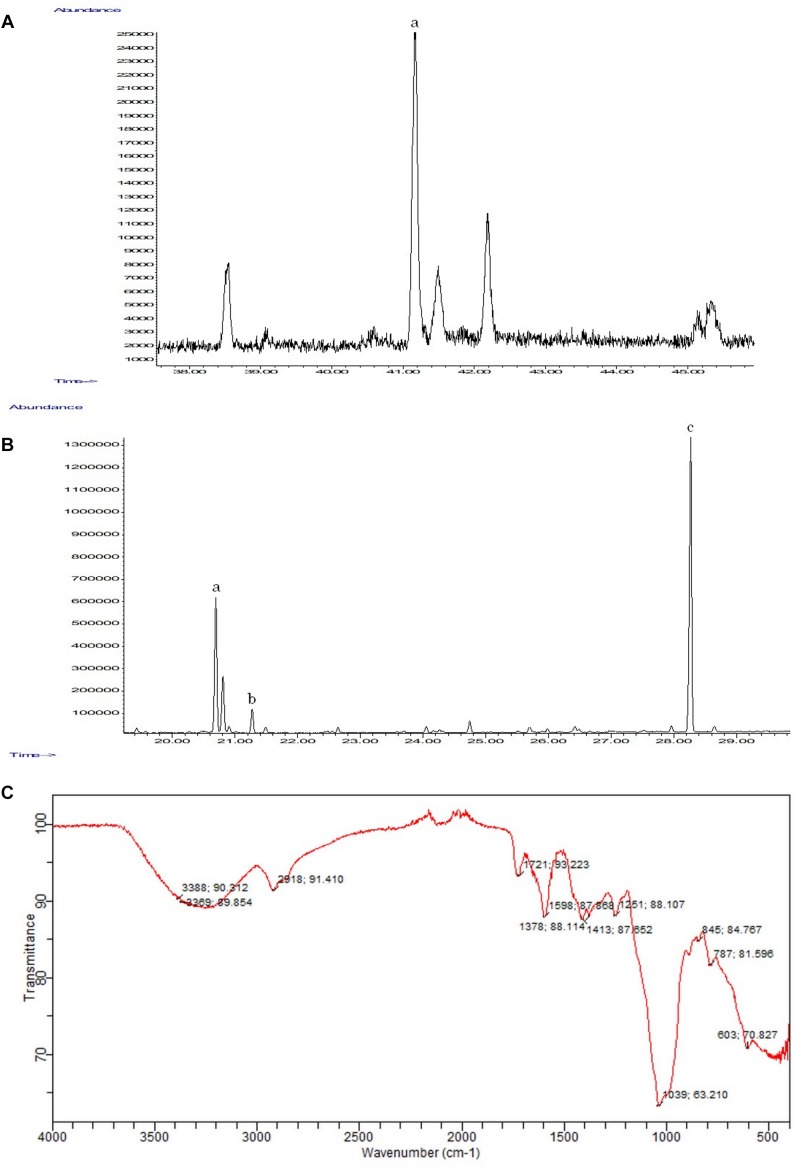
Chemical analyses performed on the ethanolic extract of the inflorescence of *E. brasiliensis*. **(A)** Extended GC-MS chromatogram of the ethanolic extract indicating the presence of γ-sitosterol (a) (rT = 41.17 min). **(B)** Expanded CG-EM chromatogram of the methylated ethanolic extract indicating the presence of linoleic (a) (rT = 20.69 min.), stearic (b) (rT = 21.27 min.), and palmitic acids (c) (rT = 28.26 min.). **(C)** FT-IR spectra of *E. brasiliensis* polysaccharides.

The GC-MS analysis confirmed the presence of the triterpene γ-sitosterol (MM = 414 g/mol) (Figure 6A), and of palmitic (MM = 270 g/mol), linoleic (MM = 292 g/mol), and stearic (MM = 298 g/mol) acids (Figure 6B). The analysis of the ethanolic extract of the inflorescence secretion by IR-TF qualitatively revealed the presence of functional groups characteristic of polysaccharides in the region of 4000–500 cm^−1^ (Figure 6C). A broad and intense band between 3500 and 3000 cm^−1^ was attributed to the presence of a hydroxyl (OH) group (Hammami et al., 2018). A less intense band at 2918.91 cm^−1^ characterized the stretch between C-H bonds (Hou et al., 2018). At 1721.93 cm^−1^, a low-intensity band attributed to the axial deformation of the C = O of enols (Silverstein et al., 2007) was detected (Figure 6C). The presence of medium-intensity absorption at 1413.87, 1378.88, and 1251.88 cm^−1^ and high-intensity absorption at 1039.63 cm^−1^ were also observed (Figure 6C), which are characteristic of the S = O bonds of sulfated esters (Silverstein et al., 2007; Webber et al., 2012).

## Discussion

Secretory structures are involved in the production of different substances, both in the vegetative and reproductive organs of plants. In flowers and inflorescences, these include such structures as idioblasts, glandular trichomes (Leite et al., 2018), colleters (Lacchia et al., 2016), laticifers (Marinho et al., 2018), osmophores, and floral and extra-floral nectaries (De Souza et al., 2005). Trichomes are classified into glandular and non-glandular trichomes. Glandular trichomes present specialized cells in the glandular head with the ability to produce, store and secrete various substances (Fahn, 1979). According to the constituents of the secretion, the glandular trichomes receive a functional denomination like the nectaries (production of nectar) and the colleters (mucilage and/or lipid-like substances secretion) (Fróes et al., 2015; Tian et al., 2017). In the Orchidaceae, researchers have previously observed the presence of nectaries (Leitão et al., 2014; Pansarin et al., 2015; Solano-Gómez et al., 2016), osmophores (Pansarin and Amaral, 2009; Millner and Baldwin, 2016; Caballero-Villalobos et al., 2017), idioblasts, and colleters (Leitão and Cortelazzo, 2008; Mayer et al., 2011; Cardoso-Gustavson et al., 2014).

Colleters have been described in at least 60 families of angiosperms (Thomas, 1991), including Apocynaceae (Appezzato-da-Glória and Estelita, 2000; Martins et al., 2013; Ribeiro et al., 2017), Bromeliaceae (Ballego-Campos and Paiva, 2018a,b), Euphorbiaceae (Vitarelli et al., 2015, 2016; Feio et al., 2016; Martins et al., 2016), Fabaceae (Paiva and Machado, 2006; Oliveira and Isaias, 2010), and Rubiaceae (Judkevich et al., 2017; Paiva-Pinheiro et al., 2019). These secretory structures are located in the vegetative and floral buds, and remain in these plant organs throughout their life. Usually, colleters produce a sticky secretion composed of a mixture of terpenes and mucilage (complex polymers of acidic or neutral polysaccharides of high molecular weight), which helps to retain water, preventing the desiccation of the meristem (Castro and Machado, 2013).

Our results confirmed the presence of colleters in the two studied species of *Elleanthus*. However, although the type of colleter was very similar in both species, the frequency of occurrence of these structures differed between the species. In *E. crinipes*, these structures are scarcer (Figure 3A) and apparently have much less secretory activity (Figure 3B), if compared with the same region in *E. brasiliensis* (Figure 2D), as could be perceived in the SEM images obtained. Histochemical analyses found evidence of the presence of mucilage and pectin in the secretion of *E. brasiliensis*. These findings confirmed the function of these structures in the studied species. Further, together with recent findings in several species from distinct lineages of Epidendroideae, the occurrence of colleters in these two tropical orchids suggests that these structures are common features of the inflorescences of tropical orchids in general (Leitão and Cortelazzo, 2008; Mayer et al., 2011; Cardoso-Gustavson et al., 2014).

In *E. brasiliensis*, the conspicuousness of the mucilaginous secretion, significant amounts of which accumulate on the plant and within the colleters, also suggests that the secretion functions as a physical barrier enclosing the flower buds and external parts of the bases of the flower tubes. Such a physical barrier would exclude nectar thieves and robbers attempting illegitimate visits to the nectaries, as well as feeding by insect herbivores.

The results of the present study revealed the presence of the triterpene γ-sitosterol and different fatty acids in the secretion of *E. brasiliensis* and the apparent absence of fungi on the surface of the flower parts of this species in SEM images. Terpenes are molecules with great structural diversity and are produced in the leaves, stems, flowers, and occasionally in the roots (Dudareva et al., 2003). In addition to playing a role in growth and development (Logan et al., 2000), as well as in pollinator attraction to plants (Pichersky and Gershenzon, 2002), these substances protect them against attacks by herbivores and pathogenic microorganisms (Paré and Tumlinson, 1999; Tholl, 2006; Cheng et al., 2007). Fatty acids constitute virtually all plant tissues and also play a role in protecting the plant organs from attack by microorganisms (McGaw et al., 2002; Kachroo and Kachroo, 2009). The recognition of pathogens by plants results in the triggering of defensive responses (Ramirez-Prado et al., 2018). These molecules and their derivatives help in these responses both in protection against bacteria (16C fatty acids) and fungi (16C and 18C fatty acids) (Kachroo and Kachroo, 2009). These findings support the idea that this secretion also functions in the protection of the inflorescences against microorganisms. In this case, the hydrated polysaccharides form a dense matrix that may act as a physical barrier, while other chemical components, such as terpenes or the fatty acids, may help to protect the inflorescence against herbivores and pathogenic microorganisms.

Through the IR-TF analysis performed, the presence of absorption bands that characterized the possible presence of polysaccharides in the secretion was observed. The presence of polysaccharides was also confirmed by the reaction with ruthenium red. However, other chromatographic analyses will be required to characterize which polysaccharide(s) are included in the secretion. Polysaccharides help in the retention of water and provide viscosity to the secretion (Toneli et al., 2005). In this way, the secretion remains adhered to the inflorescence even if it occurs in a pendent way. Although the results aided in understanding the chemical composition of the secretion, it is important to note that the analyses were performed with the secretion of several individuals. This was necessary because of the extremely low concentrations of the dry material in the individual samples, which may hinder the reproduction of these analyses in the study with this species.

Indeed, previous observations of floral visitors did not record any insect herbivore feeding on the flower parts of these species, but rather only signals of herbivory by vertebrates (e.g., birds) on the inflorescences have been seen. Therefore, this secretion is likely one of the mechanisms used to direct floral resources to the main pollinators of these orchid species, hummingbirds (Nunes et al., 2013, 2016), rather than nuisance insects. Additionally, in *E. brasiliensis* the bright and translucent mucilage in the secretion refracts the color signal of the reddish floral bracts and sepals, increasing the apparent volume of the inflorescence and reinforcing the signal to bird pollinators without advertising as much to others, such as bee pollinators (Lunau et al., 2011).

## Conclusion

Anatomical and SEM analyses revealed the presence of colleters in the inflorescences of both *Elleanthus* species studied. Although the type of colleters is very similar between these species, the low frequency of these structures’ occurrence together with an apparently reduced secretory activity results in a decrease in the production of secretion by *E. crinipes*. The presence of polysaccharides, fatty acids, and terpenes implies the role of the secretion on the hydration and protection of the inflorescences of *E. brasiliensis*. This information will contribute to the characterization of species of the family Orchidaceae, both in terms of their morphological and anatomical aspects, as well as possible plant defenses against herbivores and pathogens.

## Author Contributions

FC carried out the chemical experiments. CN was responsible for collecting the material. ML carried out the anatomical and histochemical analyses. VG assisted and interpreted in the chemical analyses. JM designed, assisted, interpreted the anatomic analysis, and supervised the work. FC wrote the manuscript and CN, ML, and JM reviewed it.

## Conflict of Interest Statement

The authors declare that the research was conducted in the absence of any commercial or financial relationships that could be construed as a potential conflict of interest.

## Abbreviations

a.s.l.: above sea level
kV: kilovolts
MM: molecular mass.
